# Global prevalence of genetic carriers for ARMC5- and KDM1A-associated primary bilateral macronodular adrenal hyperplasia: insights from a large multiethnic genomic database

**DOI:** 10.1007/s40618-026-02920-5

**Published:** 2026-05-22

**Authors:** Nipith Charoenngam, Taweesak Wannachalee

**Affiliations:** 1 Division of Endocrinology, Massachusetts General Hospital, Harvard Medical School, 50 Blossom St., Boston, MA 02114 USA; 2 Division of Endocrinology and Metabolism, Faculty of Medicine Siriraj Hospital, Mahidol University, Bangkok, Thailand

**Keywords:** Primary bilateral macronodular adrenal hyperplasia, PBMAH, gnomAD, *ARMC5*, *KDM1A*

## Abstract

**Purpose:**

To estimate the global prevalence of genetic carriers of primary bilateral macronodular adrenal hyperplasia (PBMAH)-associated variants in *ARMC5* and *KDM1A* using a large genomic database.

**Methods:**

We analyzed sequencing data from 807,162 unrelated individuals in gnomAD v4.1. Two prespecified variant-selection strategies were applied. Strict criteria included *ARMC5* and *KDM1A* variants classified as pathogenic/likely pathogenic (P/LP) in ClinVar/LOVD and germline P/LP variants reported in the literature. Liberal criteria included all strict variants plus rare predicted deleterious variants (nonsense, frameshift, loss of start codon and splice-disrupting variants predicted to be deleterious by in silico analysis) and PBMAH-reported somatic *ARMC5* variants from the literature as candidate germline susceptibility alleles. Carrier prevalence was estimated by aggregating allele frequencies of qualifying variants overall and by ancestry.

**Results:**

For *ARMC5*, estimated carrier prevalence was 37.5/100,000 under strict criteria and 64.1/100,000 under liberal criteria. Prevalence varied by ancestry, highest in East Asian individuals (strict: 111.4/100,000; liberal: 173.7/100,000) and lowest in Middle Eastern (strict: 0; liberal: 33.0/100,000) and Ashkenazi Jewish groups (strict: 6.8; liberal: 27.0/100,000). For *KDM1A*, only three variants met strict criteria; liberal criteria yielded an estimated carrier prevalence of 33.8/100,000, highest in Middle Eastern (66.0/100,000) and lowest in Finnish (6.2/100,000). Several variants were enriched in specific ancestries (e.g., *ARMC5* p.Arg454Trp in East Asians; *ARMC5* p.Arg879Trp and *KDM1A* p.Arg196AsnfsTer6 in non-Finnish Europeans).

**Conclusion:**

Population-scale data suggest PBMAH genetic susceptibility due to *ARMC5* and *KDM1A* variants may be more common than implied by clinical series and differs across ancestries, underscoring the need for improved variant curation and phenotype-linked studies.

**Supplementary Information:**

The online version contains supplementary material available at 10.1007/s40618-026-02920-5.

## Introduction

Primary bilateral macronodular adrenal hyperplasia (PBMAH) is an adrenal cause of endogenous hypercortisolism characterized by slowly progressive, bilateral, benign adrenocortical macronodules [1]. PBMAH is traditionally considered rare, but its true burden is likely underestimated because many cases are detected incidentally on adrenal imaging and present with mild or subclinical hypercortisolism. While PBMAH can occur as part of broader tumor-predisposition syndromes, most patients present with an isolated form, prompting investigation into underlying germline susceptibility [1].

Over the past decade, there have been efforts to determine the genetic basis of PBMAH. The most common established genetic etiology of PBMAH is inactivation of the tumor-suppressor Armadillo repeat-containing 5 (*ARMC5*) gene, typically via a germline heterozygous loss-of-function variant followed by a second somatic hit within adrenal nodules [2]. *ARMC5*-related PBMAH has been associated with a more pronounced degree of hypercortisolism [3]. More recently, germline lysine demethylase 1 A (*KDM1A*) inactivating variants have been identified as a rarer cause of PBMAH and are strongly associated with food-dependent Cushing syndrome (FDCS), a phenotype linked to aberrant adrenal GIP signaling [4]. Prior studies have quantified the frequencies of *ARMC5* and *KDM1A* variant carriers within PBMAH cohorts; germline *ARMC5* inactivating variants are reported in ~ 20–25% of apparently sporadic PBMAH and up to ~ 80% of familial cases, whereas germline *KDM1A* pathogenic/likely pathogenic variants are rare (< 5%) and tightly linked to food-dependent Cushing syndrome, with *KDM1A i*nactivation explaining ~ 90% of FDCS-PBMAH [1, 5, 6]. In a large multicenter series of 301 patients with *PBMAH*, 19.9% carried a germline *ARMC5* variants and 3.3% carried a germline *KDM1A* variant [6].

The advent of large-scale genomic databases now enables robust estimation of genetic disease risk across diverse populations, including conditions that may be underrecognized in routine clinical practice. PBMAH often has an insidious onset and slow tumor growth, and clinically ascertained cohorts may therefore miss many affected individuals. As such, analyses of population-scale genomic data are expected to yield higher prevalence estimates of germline carriers of PBMAH-predisposition variants than those derived from clinical case series. To enhance our understanding of PBMAH genetic risk on a global scale, we utilized the gnomAD v4.1 database [7, 8], a publicly available resource aggregating harmonized sequencing data from multiple large-scale projects, with the aim of estimating the global prevalence of genetic carriers of PBMAH-associated *ARMC5* and *KDM1A* variants and stratifying carrier frequencies by ethnic ancestry to identify potential disparities.

## Methods

### The gnomAD v4.1 database

We accessed the gnomAD v4.1 database (https://gnomad.broadinstitute.org/), which compiles population-scale sequencing data from unrelated individuals spanning multiple genetic ancestries. This database includes 730,947 exomes and 76,215 whole genomes (total 807,162 individuals) and is harmonized to the GRCh38 reference genome. gnomAD applies extensive quality-control procedures to remove low-quality samples and to flag variants with potential reliability concerns. Genetic ancestry is inferred using principal component analysis of high-quality single-nucleotide variants to define genetic clusters that are subsequently annotated based on geographic ancestry, and a random forest classifier was used to assign ancestry labels to individual samples. The methodology and code are publicly available and based on the International Genome Sample Resource [7, 8].

### Identification of potentially disease-causing variants

To estimate the global prevalence of genetic carriers of *ARMC5* and *KDM1A* variants, we applied two prespecified variant-selection approaches: the strict criteria and the liberal criteria.

Under the strict criteria, we included only variants classified as pathogenic or likely pathogenic (P/LP) in ClinVar [9] and the LOVD [10] databases (release August 23rd, 2025), because these classifications provide higher confidence that the variants are disease-causing. We excluded variants with conflicting interpretations of pathogenicity, including any variant reported as P/LP in one submission but also reported as benign, likely benign, or variant of uncertain significance (VUS) in another. We additionally included germline *ARMC5* variants designated as P/LP in a systematic review by Bouys et al. [5] and *KDM1A* P/LP variants reported in a case series by the same group [6].

Under the liberal criteria, we included all variants meeting the strict criteria and additionally included variants predicted to be deleterious with lower certainty of clinical classification. Specifically, we included null variants (nonsense, frameshift, and loss of start codon) and splice-disrupting variants predicted to be deleterious by all three in silico thresholds (SpliceAI ≥ 0.8, CADD > 20, and |pangolin| > 0.2) [11–13]. We also included *ARMC5* variants reported by Bouys et al. [5] as somatic variants because PBMAH follows a two-hit mechanism in which a germline *ARMC5* variant confers susceptibility and a second somatic alteration in adrenal tissue provides the additional inactivation required for tumor development [14]. In this setting, somatic “second-hit” variants are typically loss-of-function; therefore, variants observed as somatic events in PBMAH are likely to be functionally disruptive and were included under the liberal criteria as additional candidate disease-causing variants if present in the germline. We excluded variants with allele frequency > 1 × 10⁻⁴. The liberal criteria were designed to estimate an upper bound of carrier prevalence by incorporating additional variants with lower confidence of pathogenicity. We did not include missense variants based solely on in silico predictions because these tools can substantially overcall missense deleteriousness and could lead to overestimation of prevalence [15].

### Estimation of the prevalence of genetic carriers of *ARMC5*- and *KDM1A*-associated primary bilateral macronodular adrenal hyperplasia stratified by ethnic ancestry

To estimate the global prevalence of genetic carriers of *ARMC5*- and *KDM1A*-associated primary bilateral macronodular adrenal hyperplasia, we calculated carrier prevalence as the combined allele frequency (AF) of qualified variants, assuming it is exceedingly rare for an individual to harbor two alleles of rare variant(s). Standard errors of proportion were calculated as $\:SE=\:\sqrt{\frac{p(1-p)}{N}}$, where *p* is the estimated proportion and *N* is the sample size; SEs are shown as error bars in the bar-chart visualizations of the estimated prevalence. We applied strict variant filtering criteria (ClinVar/LOVD P/LP variants OR germline P/LP variants reported in the literature [5]) and liberal criteria (somatic variants reported in the literature [5] OR “predicted variants,” as previously mentioned) to derive lower and higher prevalence estimates, respectively. In addition, we calculated ancestry-stratified prevalence estimates and SEs within each gnomAD ethnic ancestry populations: African American/African (37,545 individuals), Admixed American (30,019 individuals), Ashkenazi Jewish (14,804 individuals), East Asian (22,448 individuals), Finnish (32,026 individuals), Middle Eastern (3,031 individuals), Non-Finnish European (590,031 individuals), South Asian (45,546 individuals) and Remaining (31,256 individuals not clustering with reference metadata) [7]. The Amish group was excluded because of insufficient sample size (456 individuals). Figures were generated using GraphPad Prism software 10.4.1 (GraphPad, La Jolla, CA, USA) and Rstudio (version 2024.9.0.375; RStudio, PBC, Boston, MA).

## Results

**Fig. 1:**
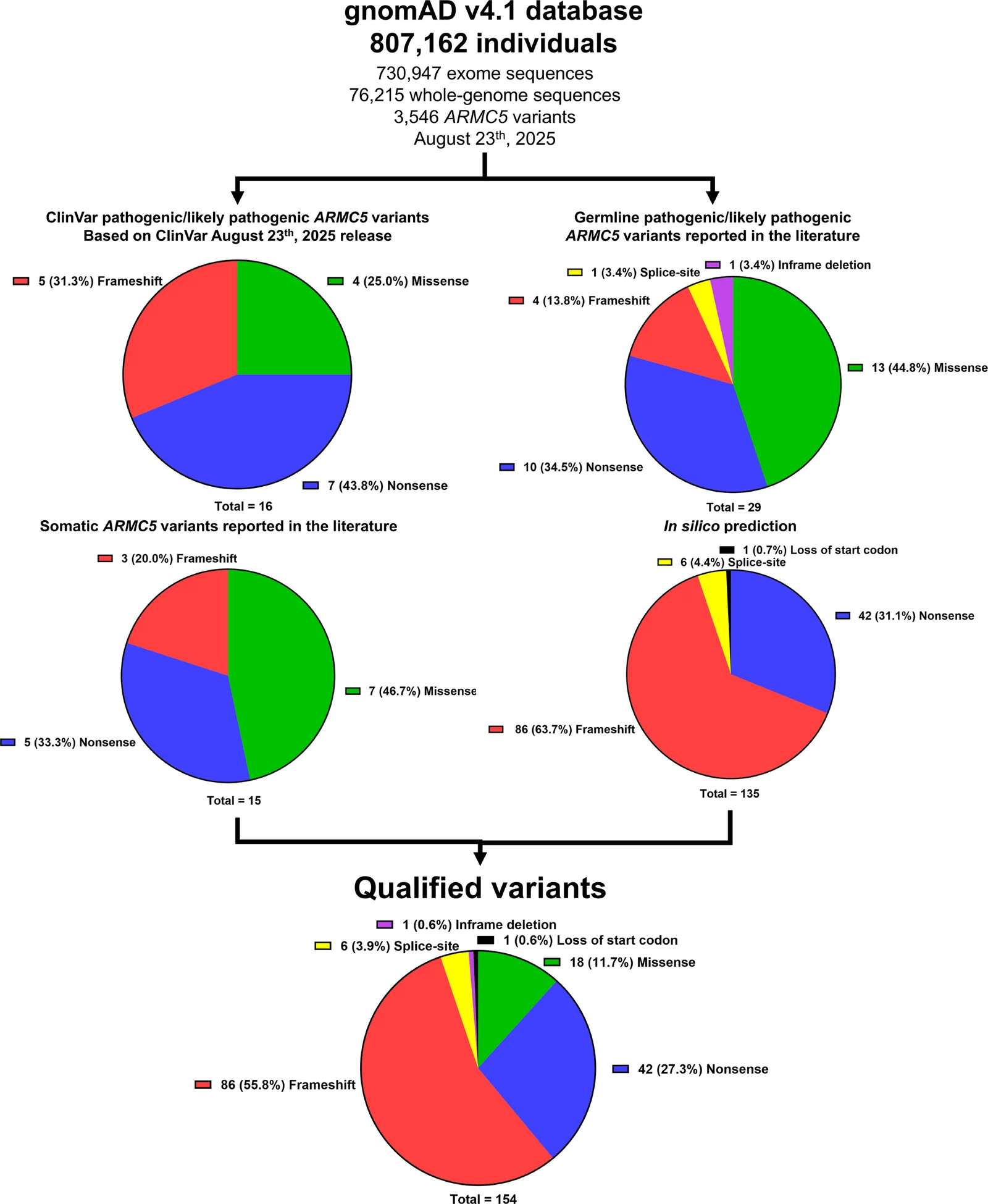
Summary of the Variant Identification Process and Proportions of Qualifying Variants by Mutation Type and Filtering Criteria. ^*^Predicted deleterious variants include disruptive splice variants predicted to be deleterious (defined by a loss-of-function flag in gnomAD v4.1 OR [SpliceAI ≥ 0.8 AND |pangolin| > 0.2]) OR frameshift OR nonsense variants, excluding ClinVar pathogenic/likely pathogenic variants/benign/likely benign variants. Predicted deleterious variants were not applicable for the *RET* gene

High-quality sequencing data were available in gnomAD for 807,162 individuals across diverse ancestral groups. Among 3,546 *ARMC5* variants in gnomAD, 16 were classified as pathogenic/likely pathogenic (P/LP) in ClinVar/LOVD, 29 as germline P/LP in the literature, 15 as somatic variants reported in the literature, and 135 were predicted to be deleterious. Figure 1 summarizes the *ARMC5* variant identification workflow and shows the distribution of variants by mutation type and filtering tier.

Among 3,423 *KDM1A* variants in gnomAD, only two met the strict criteria based on ClinVar P/LP classification (p.Arg271Ter, allele count = 1; c.352-1G > A, allele count = 1). A additional 140 *KDM1A* variants were predicted to be deleterious (81 frameshift, 2 start-loss, 36 nonsense, and 21 splice-site variants). Notably, none of the *ARMC5* or *KDM1A* variants meeting the strict criteria were observed in the 1000 Genomes Project [16]. A summary of ClinVar- and literature-classified P/LP variants is provided in Table 1, and *ARMC5* and *KDM1A* variants meeting the liberal criteria are provided in Supplementary Tables 1 and 2, respectively.

**Table 1:** List of germline *ARMC5* and *KDM1A* variants in the gnomAD v4.1 database reported in ClinVar or the literature

Gene – transcript	HGVS Consequence	Transcript Consequence	Variant type	ClinVar Germline Classification	Classification from literature	ClinVar Variation ID	rsID	Allele count
*ARMC5* – ENST00000268314.9	*p.Arg454Trp	c.1360 C > T	Missense	NR	LP	–	rs372473237	118
*ARMC5* – ENST00000268314.9	*p.Arg879Trp^β^	c.2635 C > T	Missense	VUS	LP	2,560,148	rs745559837	52
*ARMC5* – ENST00000268314.9	*p.Arg267Ter ^β^	c.799 C > T	Nonsense	P	P	144,052	rs369721476	15
*ARMC5* – ENST00000268314.9	*p.Arg173Ter ^β^	c.517 C > T	Nonsense	P	P	2,575,996	rs2082307406	14
*ARMC5* – ENST00000268314.9	*p.Arg364Ter ^β^	c.1090 C > T	Nonsense	P	P	3,048,615	rs1386368908	11
*ARMC5* – ENST00000268314.9	*p.Arg362Trp^β^	c.1084 C > T	Missense	P	P	633,679	rs1385397608	9
*ARMC5* – ENST00000268314.9	*p.Arg593Trp^α, β^	c.1777 C > T	Missense	VUS	LP	144,057	rs587777662	9
*ARMC5* – ENST00000268314.9	*p.Arg898Trp^α^	c.2692 C > T	Missense	P	LP	144,053	rs587777659	9
*ARMC5* – ENST00000268314.9	*p.Arg619Ter^β^	c.1855 C > T	Nonsense	NR	P	–	rs766717248	8
*ARMC5* – ENST00000268314.9	*p.Ile58AsnfsTer45	c.170dup	Frameshift	NR	P	–	rs951869246	8
*ARMC5* – ENST00000268314.9	*p.Pro474Ala	c.1420 C > G	Missense	VUS	LP	3,430,900	rs1298369153	7
*ARMC5* – ENST00000268314.9	p.Gly57GlufsTer80	c.170del	Frameshift	P	–	144,056	rs951869246	6
*ARMC5* – ENST00000268314.9	*p.Arg315Trp^α, β^	c.943 C > T	Missense	LP	P	3,602,762	rs2082312793	5
*ARMC5* – ENST00000268314.9	p.Ala409ThrfsTer61	c.1199_1224dup	Frameshift	P	–	1,803,212	rs2544856541	4
*ARMC5* – ENST00000268314.9	*p.Arg764Ter	c.2290 C > T	Nonsense	P	LP	1,323,417	rs778422149	3
*ARMC5* – ENST00000268314.9	p.Tyr399Ter	c.1197T > G	Nonsense	LP	–	3,580,272	rs745628267	3
*ARMC5* – ENST00000268314.9	*p.Ala610ValfsTer21	c.1827_1828dup	Frameshift	NR	P	–	–	2
*ARMC5* – ENST00000268314.9	*p.Arg654Ter^β^	c.1960 C > T	Nonsense	NR	P	–	rs1379678857	2
*ARMC5* – ENST00000268314.9	*p.Gln228Ter^β^	c.682 C > T	Nonsense	NR	P	–	–	2
*ARMC5* – ENST00000268314.9	p.Val114LeufsTer24	c.337_338dup	Frameshift	P	–	1,803,110	rs2544851883	2
*ARMC5* – ENST00000268314.9	*c.476–2 A > G	c.476–2 A > G	Splice acceptor	NR	P	–	rs1387420962	1
*ARMC5* – ENST00000268314.9	*p.Ala110ArgfsTer9^β^	c.327dup	Frameshift	P	P	3,031,151	rs1351158680	1
*ARMC5* – ENST00000268314.9	*p.Arg315Gln	c.944G > A	Missense	NR	LP	–	rs1415974570	1
*ARMC5* – ENST00000268314.9	*p.Arg362Gln	c.1085G > A	Missense	NR	LP	–	rs1172534654	1
*ARMC5* – ENST00000268314.9	*p.Arg362Pro	c.1085G > C	Missense	NR	LP	–	–	1
*ARMC5* – ENST00000268314.9	*p.Arg764Pro^β^	c.2291G > C	Missense	NR	LP	–	rs267604528	1
*ARMC5* – ENST00000268314.9	*p.Cys813ValfsTer104^β^	c.2436del	Frameshift	P	P	1,693,528	rs2142580488	1
*ARMC5* – ENST00000268314.9	*p.Gln18Ter	c.52 C > T	Nonsense	NR	P	–	–	1
*ARMC5* – ENST00000268314.9	*p.Gln86Ter^β^	c.256 C > T	Nonsense	P	P	144,054	rs587777660	1
*ARMC5* – ENST00000268314.9	*p.Glu430Ter	c.1288G > T	Nonsense	NR	P	–	–	1
*ARMC5* – ENST00000268314.9	*p.Leu548Pro^α, β^	c.1643T > C	Missense	P	LP	144,055	rs587777661	1
*ARMC5* – ENST00000268314.9	*p.Phe700del^β^	c.2097_2099del	Inframe deletion	NR	LP	–	–	1
*ARMC5* – ENST00000268314.9	*p.Pro662His^β^	c.1985 C > A	Missense	NR	LP	–	–	1
*ARMC5* – ENST00000268314.9	p.Leu596Arg	c.1787T > G	Missense	LP	–	1,339,343	rs2082338123	1
*KDM1A –* ENST00000400181.9	*p.Arg615Ter	c.1843 C > T	Nonsense	NR	P/LP	–	rs1643495733	4
*KDM1A –* ENST00000400181.9	p.Arg271Ter	c.811 C > T	Nonsense	P	–	3,366,858	–	1
*KDM1A –* ENST00000400181.9	*c.352-1G > A	c.352-1G > A	Splice acceptor	P	P/LP	3,366,857	–	1

P, pathogenic; LP, likely pathogenic; VUS, variant of uncertain significance; NR: not reported

* indicates variants reported in the literature. The rest of the variants were reported only in ClinVar or LOVD

α indicates variants with functional data. β indicates variants with evidence of a second tumoral somatic hit

## Global prevalence of genetic carriers of *ARMC5*- and *KDM1A*-associated primary bilateral macronodular adrenal hyperplasia

**Fig. 2:**
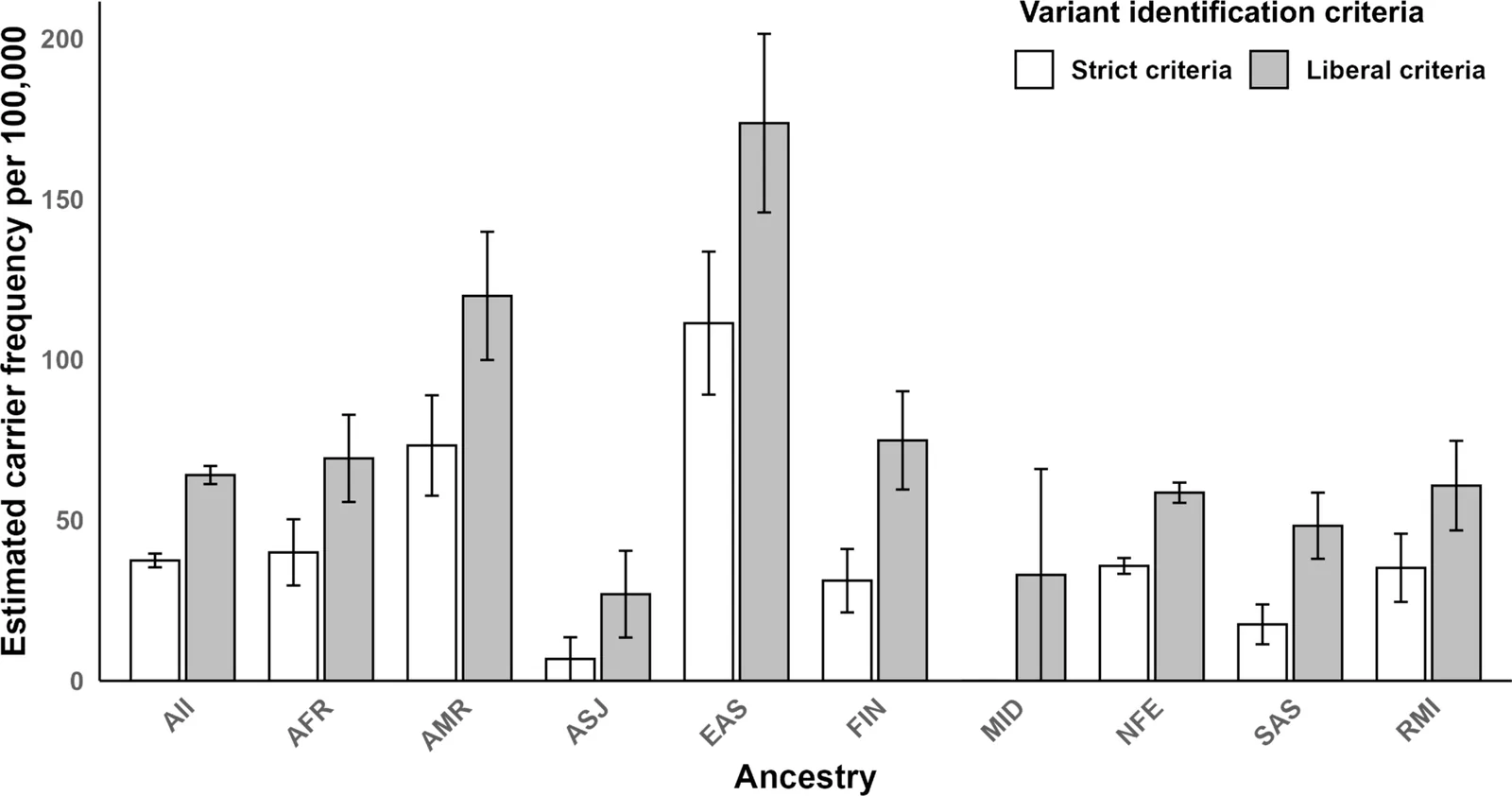
Estimated Prevalence of Genetic Carriers of Hereditary Pheochromocytoma-Paraganglioma Syndrome Among Individuals in the gnomAD Database. Data are presented as prevalence per 100,000 individuals. Dark gray bars represent prevalence estimates based on ClinVar Pathogenic/likely pathogenic (P/LP) variants only. Light gray bars represent additional predicted deleterious variants defined by (defined by a loss-of-function flag in gnomAD v4.1 OR [SpliceAI ≥ 0.8 AND |pangolin| > 0.2]) OR frameshift OR nonsense variants). Error bars represent the standard errors of the proportion (per 100,000) based on prevalence estimates that include both ClinVar P/LP variants and predicted deleterious variants. LP: Likely Pathogenic; P: Pathogenic

**Fig. 3:**
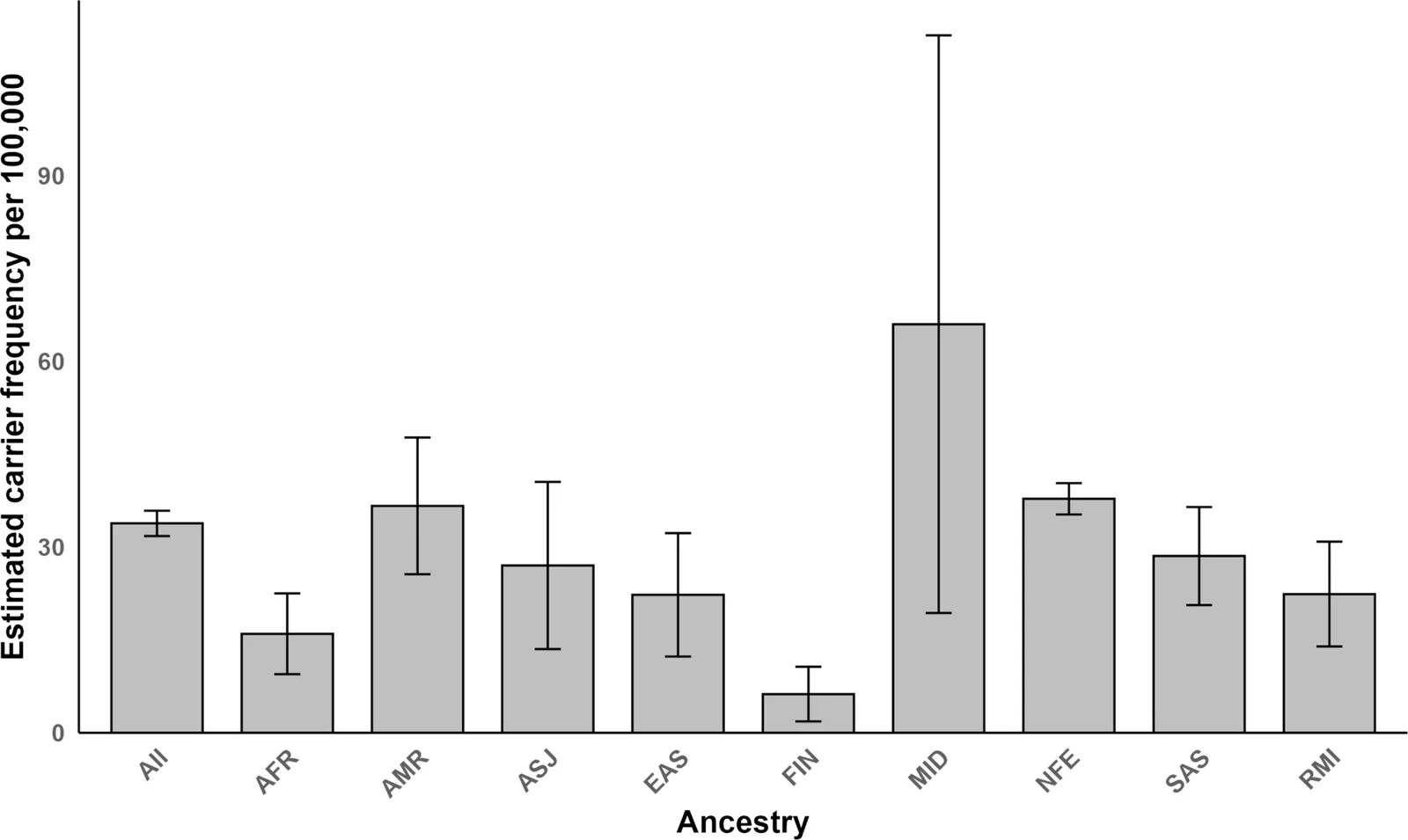
Estimated Prevalence of Genetic Carriers of Hereditary Pheochromocytoma-Paraganglioma Syndrome Stratified by Ethnic Ancestry Group. Data are presented as prevalence per 100,000 individuals. Dark gray bars represent prevalence estimates based on ClinVar Pathogenic/likely pathogenic (P/LP) variants only. Light gray bars represent additional predicted deleterious variants defined by (defined by a loss-of-function flag in gnomAD v4.1 OR [SpliceAI ≥ 0.8 AND |pangolin| > 0.2]) OR frameshift OR nonsense variants). Error bars represent the standard errors of the proportion (per 100,000) based on prevalence estimates that include both ClinVar P/LP variants and predicted deleterious variants. AFR: African/African American; AMR: Admixed American; ASJ: Ashkenazi Jewish; EAS: East Asian; FIN: Finnish; MID: Middle Eastern; NFE: Non-Finnish European; SAS: South Asian; REM: Remaining; LP: Likely Pathogenic; P: Pathogenic

Estimated carrier frequencies and corresponding standard errors for *ARMC5* and *KDM1A* are shown in Figs. 2 and 3, respectively. For *ARMC5*, using the strict criteria (ClinVar/LOVD P/LP variants or germline P/LP variants reported in the literature), the estimated carrier frequency in the overall gnomAD population was 37.5 per 100,000 (Fig. 2; Supplementary Table 3). Using the liberal criteria (strict criteria plus somatic variants reported in the literature and predicted deleterious variants from in silico analyses), the estimated carrier frequency increased to 64.1 per 100,000, providing an upper-bound estimate. The East Asian group had the highest estimated carrier frequency under both the strict (111.4 per 100,000) and liberal (173.7 per 100,000) criteria. The lowest estimates were observed in the Middle Eastern group (strict: 0 per 100,000; liberal: 33.0 per 100,000) and the Ashkenazi Jewish group (strict: 6.8 per 100,000; liberal: 27.0 per 100,000) (Fig. 2; Supplementary Table 3).

Notably, two variants, p.Arg454Trp and p.Arg879Trp (Table 1), were relatively common in the dataset, with carrier frequencies > 1 × 10⁻⁵ (7.4 × 10⁻⁵ and 3.3 × 10⁻⁵, respectively). Sensitivity analyses excluding carriers of these variants resulted in lower prevalence estimates. Under the strict criteria, the estimated prevalence (SE) decreased to 16.5 (1.4) per 100,000 overall and to 13.4 (7.7) per 100,000 in the East Asian group, suggesting that enrichment of the likely pathogenic p.Arg454Trp variant largely accounted for the higher prevalence in the East Asian group. Under the liberal criteria, the estimated prevalence likewise decreased to 43.0 (2.3) per 100,000 overall and 75.7 (18.3) per 100,000 in the East Asian group. Among variants identified in gnomAD v4.1, 18 germline *ARMC5* variants were reported in the systematic review by Bouys et al. [5] to have evidence of a second tumoral somatic hit, with a total allele count of 143. This corresponded to an estimated carrier prevalence (SE) of 17.7 (1.5) per 100,000 (Table 1). Within this subset, four variants (p.Leu548Pro, p.Arg898Trp, p.Arg315Trp and p.Arg593Trp) were supported by functional studies, with a total allele count of 24 and an estimated carrier prevalence (SE) of 2.9 (0.6) per 100,000. Ancestry-stratified carrier prevalence estimates for these sensitivity analyses are provided in Supplementary Table 4.

For *KDM1A*, only three variants met the strict criteria: two were designated pathogenic/likely pathogenic (P/LP) in ClinVar (p.Arg271Ter, allele count = 1; c.352-1G > A, allele count = 1), and one was reported in a case series by Bouys et al. [6] (p.Arg615Ter, allele count = 4) but is not reported in ClinVar. Given the limited number of qualifying variants, strict-criteria prevalence estimates were not shown. Using the liberal criteria, the estimated carrier frequency in the overall gnomAD population was 33.8 per 100,000 (Fig. 3; Supplementary Table 5). The Middle Eastern group had the highest estimated carrier frequency (66.0 per 100,000), whereas the Finnish group had the lowest (6.2 per 100,000).

Based on aggregated population allele-frequency data, qualifying variants were observed almost exclusively in the heterozygous state. One reported homozygous individual was identified for *ARMC5* p.Arg454Trp, whereas no homozygous individuals were reported for *KDM1A*. However, because individual-level data were unavailable, we could not confirm the accuracy of genotype call or evaluate the associated phenotype. Notably, several variants were enriched in specific ancestral groups in gnomAD (Tables 2 and 3). For example, the *ARMC5* p.Arg454Trp variant was enriched in East Asian individuals (allele count = 20), and *ARMC5* p.Arg879Trp was enriched in the non-Finnish European group (allele count = 45) (Table 2). Similarly, the *KDM1A* p.Arg196AsnfsTer6 variant was enriched in the non-Finnish European group (allele count = 49) (Table 3).

**Table 2:** List of *ARMC5* variants in the gnomAD v4.1 database enriched in specific ethnic ancestry groups

Gene – transcript	Ethnic ancestry	HGVS Consequence	Transcript Consequence	Variant type	rsID	Allele count within ancestry group	GroupMax FAF frequency (per 100,000)
*ARMC5* – ENST00000268314.9	AFR	*p.Arg267Ter	c.799 C > T	Nonsense	rs369721476	3	1.06
*ARMC5* – ENST00000268314.9	AFR	*p.Arg593Trp	c.1777 C > T	Missense	rs587777662	3	1.06
*ARMC5* – ENST00000268314.9	AFR	p.Tyr697Ter	c.2091 C > A	Nonsense	rs967154363	3	1.06
*ARMC5* – ENST00000268314.9	AFR	*p.Ile58AsnfsTer45	c.170dup	Frameshift	rs951869246	2	0.46
*ARMC5* – ENST00000268314.9	AFR	*p.Tyr399Ter	c.1197T > G	Nonsense	rs745628267	2	0.44
*ARMC5* – ENST00000268314.9	AMR	p.Gln21Ter	c.61 C > T	Nonsense	rs762503656	4	2.63
*ARMC5* – ENST00000268314.9	AMR	p.Pro706ArgfsTer29	c.2115_2116dup	Frameshift	rs761413187	4	2.26
*ARMC5* – ENST00000268314.9	AMR	*p.Gly57GlufsTer80	c.170del	Frameshift	rs951869246	2	0.63
*ARMC5* – ENST00000268314.9	EAS	*p.Arg454Trp	c.1360 C > T	Missense	rs372473237	20	29.78
*ARMC5* – ENST00000268314.9	EAS	p.Trp679ThrfsTer11	c.2034_2040del	Frameshift	rs764430983	8	8.84
*ARMC5* – ENST00000268314.9	EAS	p.Met1?	c.1 A > G	Start lost	rs1169096987	2	0.81
*ARMC5* – ENST00000268314.9	NFE	*p.Arg879Trp	c.2635 C > T	Missense	rs745559837	45	2.98
*ARMC5* – ENST00000268314.9	NFE	p.Arg841His	c.2522G > A	Missense	rs1279675410	10	0.43
*ARMC5* – ENST00000268314.9	NFE	p.Cys713ValfsTer23	c.2128_2132dup	Frameshift	rs1423480908	10	0.43
*ARMC5* – ENST00000268314.9	NFE	*p.Arg364Ter	c.1090 C > T	Nonsense	rs1386368908	9	0.36
*ARMC5* – ENST00000268314.9	NFE	*p.Arg362Trp	c.1084 C > T	Missense	rs1385397608	8	0.29
*ARMC5* – ENST00000268314.9	NFE	*p.Arg898Trp^β^	c.2692 C > T	Missense	rs587777659	6	0.19
*ARMC5* – ENST00000268314.9	NFE	p.Met641TrpfsTer51	c.1920del	Frameshift	rs1567377765	6	0.18
*ARMC5* – ENST00000268314.9	NFE	*p.Pro474Ala	c.1420 C > G	Missense	rs1298369153	5	0.13
*ARMC5* – ENST00000268314.9	NFE	*p.Ala409ThrfsTer61	c.1199_1224dup	Frameshift	–	4	0.08
*ARMC5* – ENST00000268314.9	NFE	p.Glu148Ter	c.442G > T	Nonsense	–	4	0.08
*ARMC5* – ENST00000268314.9	NFE	p.Leu650PhefsTer42	c.1948del	Frameshift	–	4	0.08
*ARMC5* – ENST00000268314.9	NFE	p.Pro241AlafsTer34	c.720_721del	Frameshift	–	4	0.08
*ARMC5* – ENST00000268314.9	NFE	p.Tyr626Ter	c.1878 C > G	Nonsense	–	4	0.08
*ARMC5* – ENST00000268314.9	NFE	p.Val239AlafsTer36	c.716_717del	Frameshift	–	4	0.08
*ARMC5* – ENST00000268314.9	NFE	*p.Arg764Ter	c.2290 C > T	Nonsense	rs778422149	3	0.07
*ARMC5* – ENST00000268314.9	NFE	p.Ala492ArgfsTer46	c.1473dup	Frameshift	–	3	0.07
*ARMC5* – ENST00000268314.9	NFE	p.Ala692GlyfsTer9	c.2074_2075insGTAG	Frameshift	rs754210813	3	0.07
*ARMC5* – ENST00000268314.9	NFE	p.Arg793Ter	c.2377 C > T	Nonsense	rs1489499125	3	0.07
*ARMC5* – ENST00000268314.9	NFE	*p.Ala610ValfsTer21	c.1827_1828dup	Frameshift	–	2	0.03
*ARMC5* – ENST00000268314.9	NFE	*p.Arg315Trp	c.943 C > T	Missense	rs2082312793	2	0.03
*ARMC5* – ENST00000268314.9	NFE	*p.Gln228Ter	c.682 C > T	Nonsense	–	2	0.03
*ARMC5* – ENST00000268314.9	NFE	*p.Val114LeufsTer24	c.337_338dup	Frameshift	–	2	0.03
*ARMC5* – ENST00000268314.9	NFE	c.1370 + 1G > A	c.1370 + 1G > A	Splice donor	–	2	0.03
*ARMC5* – ENST00000268314.9	NFE	p.Gln214Ter	c.640 C > T	Nonsense	–	2	0.03
*ARMC5* – ENST00000268314.9	NFE	p.Gln709Ter	c.2125 C > T	Nonsense	–	2	0.03
*ARMC5* – ENST00000268314.9	NFE	p.Glu325Ter	c.973G > T	Nonsense	–	2	0.03
*ARMC5* – ENST00000268314.9	NFE	p.Glu517Ter	c.1549G > T	Nonsense	–	2	0.03
*ARMC5* – ENST00000268314.9	NFE	p.Glu667SerfsTer25	c.1999del	Frameshift	–	2	0.03
*ARMC5* – ENST00000268314.9	NFE	p.Glu907ArgfsTer10	c.2719del	Frameshift	–	2	0.03
*ARMC5* – ENST00000268314.9	NFE	p.Leu70TyrfsTer67	c.208del	Frameshift	–	2	0.03
*ARMC5* – ENST00000268314.9	NFE	p.Pro675GlnfsTer17	c.2024del	Frameshift	rs761103963	2	0.03
*ARMC5* – ENST00000268314.9	NFE	p.Ser344ProfsTer31	c.1029del	Frameshift	rs1567375706	2	0.03
*ARMC5* – ENST00000268314.9	NFE	p.Ser689LeufsTer3	c.2064del	Frameshift	rs1418564135	2	0.03
*ARMC5* – ENST00000268314.9	NFE	p.Thr643ProfsTer11	c.1923_1926dup	Frameshift	–	2	0.03
*ARMC5* – ENST00000268314.9	NFE	p.Trp668Ter	c.2003G > A	Nonsense	–	2	0.03
*ARMC5* – ENST00000268314.9	NFE	p.Val114CysfsTer23	c.340del	Frameshift	–	2	0.03
*ARMC5* – ENST00000268314.9	SAS	p.Pro633HisfsTer59	c.1898del	Frameshift	rs768159977	4	1.43
*ARMC5* – ENST00000268314.9	SAS	*p.Arg173Ter	c.517 C > T	Nonsense	rs2082307406	3	0.87
*ARMC5* – ENST00000268314.9	SAS	*p.Arg619Ter	c.1855 C > T	Nonsense	rs766717248	2	0.37

AFR: African/African American; AMR: Admixed American; EAS: East Asian; MID: Middle Eastern; NFE: Non-Finnish European; SAS: South Asian

* indicates variants reported in the literature or ClinVar/LOVD. All other variants were predicted to be potentially deleterious by in silico analysis. Groupmax FAF is defined as the highest filtering allele frequency observed among all gnomAD continental ancestry groups, expressed per 100,000 individuals

**Table 3:** List of *KDM1A* variants in the gnomAD v4.1 database enriched in specific ethnic ancestry groups

Gene – transcript	Ethnic ancestry	HGVS Consequence	Transcript Consequence	Variant type	rsID	Allele count within ancestry group	GroupMax FAF frequency (per 100,000)
*KDM1A –* ENST00000400181.9	AFR	c.1849_1867 + 39dup	c.1849_1867 + 39dup	Nonsense	rs1557596078	4	1.75
*KDM1A –* ENST00000400181.9	NFE	p.Arg196AsnfsTer6	c.587_590del	Frameshift	–	49	4.95
*KDM1A –* ENST00000400181.9	NFE	p.Arg844Ter	c.2530 C > T	Nonsense	–	12	0.54
*KDM1A –* ENST00000400181.9	NFE	p.Trp815Ter	c.2445G > A	Nonsense	rs1358867166	10	0.43
*KDM1A –* ENST00000400181.9	NFE	p.Ile833TyrfsTer19	c.2496_2497insT	Frameshift	–	8	0.38
*KDM1A –* ENST00000400181.9	NFE	p.Arg839PhefsTer14	c.2510_2513dup	Frameshift	–	6	0.26
*KDM1A –* ENST00000400181.9	NFE	p.Phe837SerfsTer29	c.2510_2513del	Frameshift	–	3	0.1
*KDM1A –* ENST00000400181.9	NFE	p.Ile833PhefsTer34	c.2497del	Frameshift	–	3	0.09
*KDM1A –* ENST00000400181.9	NFE	p.Arg336Ter	c.1006 C > T	Nonsense	rs1369524188	4	0.08
*KDM1A –* ENST00000400181.9	NFE	p.Arg615Ter	c.1843 C > T	Nonsense	rs1643495733	4	0.08
*KDM1A –* ENST00000400181.9	NFE	p.Arg819Ter	c.2455 C > T	Nonsense	rs746295431	4	0.08
*KDM1A –* ENST00000400181.9	NFE	p.Leu854TrpfsTer29	c.2561del	Frameshift	rs1643675950	4	0.08
*KDM1A –* ENST00000400181.9	NFE	p.Lys480AsnfsTer12	c.1440_1443del	Frameshift	rs763785057	4	0.08
*KDM1A –* ENST00000400181.9	NFE	p.Glu32Ter	c.94G > T	Nonsense	–	3	0.07
*KDM1A –* ENST00000400181.9	NFE	p.Gly623Ter	c.1867G > T	Nonsense	–	3	0.07
*KDM1A –* ENST00000400181.9	NFE	p.Lys246Ter	c.736 A > T	Nonsense	–	3	0.07
*KDM1A –* ENST00000400181.9	NFE	p.Arg839PhefsTer30	c.2509_2513dup	Frameshift	–	2	0.04
*KDM1A –* ENST00000400181.9	NFE	p.Arg750Ter	c.2248 C > T	Nonsense	–	2	0.03
*KDM1A –* ENST00000400181.9	NFE	p.Gln871ProfsTer54	c.2611dup	Frameshift	–	2	0.03
*KDM1A –* ENST00000400181.9	NFE	p.Leu651ArgfsTer4	c.1952del	Frameshift	rs1242225620	2	0.03
*KDM1A –* ENST00000400181.9	NFE	p.Pro869LeufsTer14	c.2606del	Frameshift	rs1196220949	2	0.03
*KDM1A –* ENST00000400181.9	NFE	p.Ser569AsnfsTer5	c.1705_1706del	Frameshift	–	2	0.03
*KDM1A –* ENST00000400181.9	NFE	p.Val771GlyfsTer7	c.2312_2313del	Frameshift	–	2	0.03
*KDM1A –* ENST00000400181.9	SAS	p.Arg202Ter	c.604 C > T	Nonsense	rs1341951118	2	0.37

AFR: African/African American; AMR: Admixed American; EAS: East Asian; MID: Middle Eastern; NFE: Non-Finnish European; SAS: South Asian

All variants were predicted to be potentially deleterious by in silico analysis. Groupmax FAF is defined as the highest filtering allele frequency observed among all gnomAD continental ancestry groups, expressed per 100,000 individuals

## Discussion

PBMAH is traditionally considered a very rare entity, and estimating its true frequency is challenging. Epidemiologic studies of endogenous Cushing syndrome (CS) report an overall incidence of approximately 1.8–3.2 cases per million per year and a prevalence of 57–79 per million [17]; adrenal causes account for about 20–30% of CS, and PBMAH usually represents < 2% of CS cases, implying a population prevalence in the low single digits per million [18, 19]. In a multicenter study of 352 consecutive index patients with PBMAH, 52 (14.8%) carried germline pathogenic *ARMC5* variants and had more marked cortisol excess, larger adrenal size, and were more often surgically or medically treated than non-carriers [20].

Another approach uses prevalence estimates of adrenal incidentalomas, which are found in roughly 1–5% of abdominal CT scans, and approximately 10–20% are bilateral [21]. In one study, PBMAH accounted for ~ 4% of all adrenal incidentalomas but nearly one-third of bilateral lesions with subtle hypercortisolism [22]. Recent systematic adrenal imaging data further suggest that bilateral macronodular adrenocortical disease (BMAD) may be more common than estimates derived solely from clinically overt Cushing syndrome. In a systematic adrenal imaging screening study of 25,356 unselected adults, adrenal tumors were identified in 1.4% of participants, and a subsequent analysis found bilateral lesions in 9.1% of adrenal nodules [23]. Although these studies were not designed to directly measure BMAD prevalence, they suggest that the true population prevalence may plausibly lie in the range of 5–75 per 100,000, substantially higher than CS-based estimates in the low single digits per million [24].

In this study, we leveraged population-scale genomic data from gnomAD (*n* = 807,162) to estimate the prevalence of germline carriers of *ARMC5* and *KDM1A* variants anticipated to disrupt gene function and confer susceptibility to PBMAH. For *ARMC5*, the estimated carrier prevalence was 37.5 per 100,000 under prespecified strict criteria restricted to ClinVar/LOVD and literature-designated pathogenic/likely pathogenic variants; this estimate increased to 64.1 per 100,000 under liberal criteria that additionally incorporated rare predicted loss-of-function or splice-disrupting variants and PBMAH-reported somatic variants from the literature as candidate germline susceptibility alleles. Carrier prevalence varied substantially by ancestry, with the highest estimates observed among individuals of East Asian ancestry and the lowest among the Middle Eastern and Ashkenazi Jewish groups. For *KDM1A*, only two variants met strict ClinVar/LOVD criteria, whereas liberal criteria yielded an estimated carrier prevalence of 33.8 per 100,000, with similarly marked ancestry-specific differences. This apparent rarity is consistent with a multicenter study showing that germline *KDM1A* P/LP variants were uncommon among PBMAH cases (< 5% of index cases) and concentrated within a clinically distinctive subset associated with food-dependent CS [20]. Collectively, these results indicate that population-level germline susceptibility to PBMAH may be more common than implied by clinically ascertained series, consistent with underrecognition of PBMAH and/or milder phenotypic expression among genetically predisposed individuals.

In addition, incomplete penetrance and variable expressivity may contribute to the discrepancy between population-based carrier prevalence and clinically recognized PBMAH. Disease expression may be influenced by phenotype-modifying genetic background and epigenomic regulation [19]. Integrated genomic studies of *ARMC5*-mutated PBMAH have identified distinctive molecular features, including CpG-island hypermethylation and recurrent copy-neutral loss of heterozygosity at chromosome 16p, consistent with acquired regulatory changes that may modulate nodular growth and steroidogenesis [19, 25]. Additionally, heterogeneity in aberrant G protein-coupled receptor signaling, including autocrine/paracrine mechanisms (e.g., locally produced ACTH and serotonin), may further contribute to variability in cortisol secretion and clinical detection among carriers [19].

The marked discrepancy between strict and liberal prevalence estimates may also reflect gaps in case ascertainment and genetic testing for PBMAH, which in turn limit variant submission and curation in clinical variant interpretation databases (e.g., ClinVar/LOVD) and the published literature. For *ARMC5*, most variants contributing to our prevalence estimates were captured only under the liberal criteria, with > 75% predicted to be deleterious but not previously curated in ClinVar/LOVD. This issue was even more prominent for *KDM1A*, for which only two ClinVar/LOVD P/LP variants were observed in gnomAD despite 140 additional rare variants predicted to be deleterious, suggesting that many uncurated yet plausibly deleterious variants in the population remain absent from these databases and consistent with underrecognition and underreporting of PBMAH-associated variation.

Several limitations should be acknowledged. First, gnomAD lacks linked phenotype data, precluding assessment of genotype-phenotype associations, age at presentation or penetrance; therefore, our estimates represent the population frequency of carriers of qualifying variants, not the prevalence of clinically manifest PBMAH. Second, gnomAD may underrepresent individuals with severe diseases because samples are drawn from heterogeneous studies with variable ascertainment and may exclude some individuals with serious pediatric or syndromic conditions. Third, although our strict criteria prioritize variants with higher-confidence clinical classification, our liberal criteria intentionally incorporate predicted loss-of-function and splice-disrupting variants as well as PBMAH-reported somatic variants as candidate germline susceptibility alleles; this strategy provides an upper-bound estimate but may overestimate true pathogenic carrier prevalence because not all predicted disruptive variants are truly disease-causing. Finally, ancestry-stratified estimates are less precise in smaller gnomAD groups, particularly the Middle Eastern, Ashkenazi Jewish, and East Asian populations.

Despite these limitations, our findings have important clinical and research implications. The population frequency of qualifying *ARMC5* and *KDM1A* variants, together with the marked ancestry-specific differences, suggests that genetic susceptibility to PBMAH may be more common than indicated by clinically ascertained case series, potentially reflecting underrecognition of PBMAH and/or incomplete penetrance. These results support consideration of germline *ARMC5* (and, where appropriate, *KDM1A*) testing in patients evaluated for PBMAH, particularly in the setting of bilateral adrenal nodularity with overt or subtle hypercortisolism [26]. More broadly, the gap between strict and liberal estimates underscores the need for improved case ascertainment, expanded access to genetic testing, functional validation of candidate variants, and timely submission and curation of PBMAH-associated variants in clinical variant interpretation databases. Future studies that link large-scale genomic data to detailed phenotypes will be essential to refine prevalence and penetrance estimates, clarify genotype-phenotype associations, and determine how ancestry-specific variant distributions translate into clinical risk.

## Conclusion

In conclusion, leveraging sequencing data from 807,162 individuals in gnomAD v4.1, we provide population-based estimates of germline carrier frequencies for *ARMC5*- and *KDM1A*-associated PBMAH susceptibility variants using prespecified strict and liberal variant-selection frameworks. We found that *ARMC5* carrier prevalence was 37.5 per 100,000 under strict criteria and 64.1 per 100,000 under liberal criteria, while *KDM1A* variants were rare under strict ClinVar/LOVD classification but yielded an estimated 33.8 per 100,000 carriers under liberal criteria, with substantial ancestry-specific variation and enrichment of certain variants in particular populations. Together, these findings suggest that genetic susceptibility to PBMAH may be more common than implied by clinically ascertained series, consistent with underrecognition and/or incomplete penetrance, and they underscore the need for improved variant curation, functional validation, and phenotype-linked genomic studies to refine penetrance and translate ancestry-informed variant distributions into clinically actionable risk assessment.

## Supplementary Information

Below is the link to the electronic supplementary material.

Supplementary Material 1
