# Supplementary material for: Global prevalence of genetic carriers for ARMC5- and KDM1A-associated primary bilateral macronodular adrenal hyperplasia: insights from a large multiethnic genomic database
Source: J Endocrinol Invest. 2026 May 22;49(9):2291–303. doi: 10.1007/s40618-026-02920-5 (PMC13498619; doi:10.1007/s40618-026-02920-5)
Supplement: Supplementary file 1 [file 40618_2026_2920_MOESM1_ESM.docx]

**6Supplementary Table 1**. ARMC5 variants reported as somatic variants in the literature or predicted to be potentially deleterious by in silico tools, but not reported as germline pathogenic or likely pathogenic in ClinVar/LOVD or the literature.

| **HGVS Consequence** | **Transcript Consequence** | **Variant type** | **ClinVar Variation ID** | **rsID** | **Allele count** |
| --- | --- | --- | --- | --- | --- |
| *p.Arg841His | c.2522G>A | Missense | - | rs1279675410 | 12 |
| p.Cys713ValfsTer23 | c.2128_2132dup | Frameshift | - | rs1423480908 | 10 |
| p.Trp679ThrfsTer11 | c.2034_2040del | Frameshift | - | rs764430983 | 8 |
| p.Met641TrpfsTer51 | c.1920del | Frameshift | 591500 | rs1567377765 | 7 |
| p.Glu148Ter | c.442G>T | Nonsense | - | - | 5 |
| p.Pro633HisfsTer59 | c.1898del | Frameshift | - | rs768159977 | 5 |
| p.Pro706ArgfsTer29 | c.2115_2116dup | Frameshift | - | rs761413187 | 5 |
| p.Gln21Ter | c.61C>T | Nonsense | - | rs762503656 | 4 |
| p.Leu650PhefsTer42 | c.1948del | Frameshift | - | - | 4 |
| p.Pro241AlafsTer34 | c.720_721del | Frameshift | - | - | 4 |
| p.Tyr626Ter | c.1878C>G | Nonsense | - | - | 4 |
| p.Tyr697Ter | c.2091C>A | Nonsense | - | rs967154363 | 4 |
| p.Val239AlafsTer36 | c.716_717del | Frameshift | - | - | 4 |
| p.Ala492ArgfsTer46 | c.1473dup | Frameshift | - | - | 3 |
| p.Ala692GlyfsTer9 | c.2074_2075insGTAG | Frameshift | - | rs754210813 | 3 |
| p.Arg793Ter | c.2377C>T | Nonsense | - | rs1489499125 | 3 |
| p.Leu70ProfsTer33 | c.208dup | Frameshift | - | - | 3 |
| p.Pro675GlnfsTer17 | c.2024del | Frameshift | - | rs761103963 | 3 |
| p.Ser112ProfsTer25 | c.333del | Frameshift | - | - | 3 |
| *p.Gln408Ter | c.1222C>T | Nonsense | - | rs1367418052 | 2 |
| p.Asn576MetfsTer53 | c.1727_1730del | Frameshift | - | - | 2 |
| p.Cys575Ter | c.1724_1725del | Frameshift | - | - | 2 |
| p.Cys579AlafsTer44 | c.1734_1755del | Frameshift | - | - | 2 |
| p.Gln214Ter | c.640C>T | Nonsense | - | - | 2 |
| p.Gln709Ter | c.2125C>T | Nonsense | - | - | 2 |
| p.Glu325Ter | c.973G>T | Nonsense | - | - | 2 |
| p.Glu517Ter | c.1549G>T | Nonsense | - | - | 2 |
| p.Glu667SerfsTer25 | c.1999del | Frameshift | - | - | 2 |
| p.Glu907ArgfsTer10 | c.2719del | Frameshift | - | - | 2 |
| p.Leu70TyrfsTer67 | c.208del | Frameshift | - | - | 2 |
| p.Met1? | c.1A>G | Loss-of-start-codon | - | rs1169096987 | 2 |
| p.Ser26Ter | c.77C>A | Nonsense | - | rs1221666606 | 2 |
| p.Ser344ProfsTer31 | c.1029del | Frameshift | - | rs1567375706 | 2 |
| p.Ser689LeufsTer3 | c.2064del | Frameshift | - | rs1418564135 | 2 |
| p.Thr643ProfsTer11 | c.1923_1926dup | Frameshift | - | - | 2 |
| p.Trp668Ter | c.2003G>A | Nonsense | - | - | 2 |
| p.Val114CysfsTer23 | c.340del | Frameshift | - | - | 2 |
|  | c.1370+1G>A | Splice donor | - | - | 2 |
| *p.Ala110ProfsTer27 | c.327del | Frameshift | - | - | 1 |
| *p.Ala144ArgfsTer16 | c.430del | Frameshift | - | - | 1 |
| *p.Ala224Glu | c.671C>A | Missense | - | - | 1 |
| *p.Ala83Pro | c.247G>C | Missense | - | - | 1 |
| *p.Ala83Pro | c.247G>C | Missense | - | rs758286564 | 1 |
| *p.Leu40Ter | c.118del | Frameshift | - | - | 1 |
| *p.Lys31Ter | c.91A>T | Nonsense | - | - | 1 |
| p.Ala100ProfsTer10 | c.297_322del | Frameshift | - | - | 1 |
| p.Ala110ProfsTer27 | c.328del | Frameshift | - | - | 1 |
| p.Ala590GlyfsTer10 | c.1769_1779del | Frameshift | - | - | 1 |
| p.Ala641GlyfsTer12 | c.1921dup | Frameshift | - | - | 1 |
| p.Ala658SerfsTer42 | c.1970dup | Frameshift | - | - | 1 |
| p.Ala702ArgfsTer15 | c.2103del | Frameshift | - | - | 1 |
| p.Ala743ProfsTer174 | c.2226del | Frameshift | - | - | 1 |
| p.Ala766ProfsTer151 | c.2296del | Frameshift | - | - | 1 |
| p.Ala770GlyfsTer106 | c.2296_2308dup | Frameshift | - | rs779975406 | 1 |
| p.Ala776GlyfsTer96 | c.2326dup | Frameshift | - | - | 1 |
| p.Ala882GlyfsTer30 | c.2645_2660del | Frameshift | - | - | 1 |
| p.Ala88ArgfsTer48 | c.262del | Frameshift | - | - | 1 |
| p.Ala88GlyfsTer3 | c.263_264del | Frameshift | - | - | 1 |
| p.Ala931GlnfsTer18 | c.2791del | Frameshift | - | - | 1 |
| p.Arg221ValfsTer34 | c.661del | Frameshift | - | - | 1 |
| p.Arg366ProfsTer37 | c.1090_1096dup | Frameshift | - | rs1445646111 | 1 |
| p.Arg572AlafsTer58 | c.1714del | Frameshift | - | - | 1 |
| p.Arg673ProfsTer64 | c.2018_2019del | Frameshift | - | - | 1 |
| p.Arg879GlyfsTer38 | c.2635del | Frameshift | - | - | 1 |
| p.Arg93Ter | c.277C>T | Nonsense | - | rs763121282 | 1 |
| p.Asn20ThrfsTer12 | c.59del | Frameshift | - | - | 1 |
| p.Cys813TrpfsTer104 | c.2439del | Frameshift | - | - | 1 |
| p.Cys894Ter | c.2682C>A | Nonsense | - | - | 1 |
| p.Gln208Ter | c.622C>T | Nonsense | - | rs760562692 | 1 |
| p.Gln276Ter | c.826C>T | Nonsense | - | rs747051184 | 1 |
| p.Gln449HisfsTer13 | c.1345_1346dup | Frameshift | - | - | 1 |
| p.Gln56Ter | c.166C>T | Nonsense | - | - | 1 |
| p.Gln672Ter | c.2014C>T | Nonsense | - | - | 1 |
| p.Gln683Ter | c.2047C>T | Nonsense | - | rs1239313088 | 1 |
| p.Gln72Ter | c.214C>T | Nonsense | - | rs2082271953 | 1 |
| p.Gln759Ter | c.2275C>T | Nonsense | - | rs2082358993 | 1 |
| p.Glu142GlyfsTer45 | c.424dup | Frameshift | - | rs1567373997 | 1 |
| p.Glu142TyrfsTer46 | c.420_423dup | Frameshift | - | - | 1 |
| p.Glu25ArgfsTer16 | c.73del | Frameshift | - | - | 1 |
| p.Glu275SerfsTer52 | c.822_832del | Frameshift | - | - | 1 |
| p.Glu330SerfsTer45 | c.988del | Frameshift | - | - | 1 |
| p.Glu358Ter | c.1070dup | Frameshift | - | rs1567375764 | 1 |
| p.Glu433AspfsTer8 | c.1299_1300del | Frameshift | - | - | 1 |
| p.Glu442ArgfsTer19 | c.1324del | Frameshift | - | - | 1 |
| p.Glu737Ter | c.2209G>T | Nonsense | - | - | 1 |
| p.Glu928Ter | c.2782G>T | Nonsense | - | - | 1 |
| p.Gly418TrpfsTer6 | c.1251dup | Frameshift | - | - | 1 |
| p.Gly7Ter | c.19G>T | Nonsense | - | - | 1 |
| p.Gly99GlufsTer38 | c.296del | Frameshift | - | - | 1 |
| p.His618AlafsTer17 | c.1851dup | Frameshift | - | - | 1 |
| p.His653ProfsTer39 | c.1958del | Frameshift | - | - | 1 |
| p.His694LeufsTer31 | c.2081_2118del | Frameshift | - | - | 1 |
| p.His883CysfsTer30 | c.2643_2646dup | Frameshift | - | rs2082364128 | 1 |
| p.His883LeufsTer28 | c.2646_2647del | Frameshift | - | rs1567379542 | 1 |
| p.Leu131Ter | c.392T>A | Nonsense | - | - | 1 |
| p.Leu254ArgfsTer3 | c.761del | Frameshift | - | - | 1 |
| p.Leu654CysfsTer38 | c.1960del | Frameshift | - | - | 1 |
| p.Leu827ArgfsTer90 | c.2480del | Frameshift | - | rs754932897 | 1 |
| p.Leu916Ter | c.2746del | Frameshift | - | - | 1 |
| p.Lys31ArgfsTer10 | c.92del | Frameshift | - | - | 1 |
| p.Phe77ArgfsTer60 | c.223_224dup | Frameshift | - | - | 1 |
| p.Pro316HisfsTer12 | c.947_950del | Frameshift | - | - | 1 |
| p.Pro33GlnfsTer8 | c.98del | Frameshift | - | - | 1 |
| p.Pro388LeufsTer73 | c.1163del | Frameshift | - | - | 1 |
| p.Pro493ArgfsTer51 | c.1478del | Frameshift | - | - | 1 |
| p.Pro822HisfsTer95 | c.2465del | Frameshift | - | - | 1 |
| p.Ser533ProfsTer11 | c.1597del | Frameshift | 591035 | rs1567377180 | 1 |
| p.Ser650AlafsTer10 | c.1948del | Frameshift | - | rs1440446878 | 1 |
| p.Ser796Ter | c.2387C>A | Nonsense | - | - | 1 |
| p.Thr715LeufsTer2 | c.2142del | Frameshift | - | - | 1 |
| p.Trp459Ter | c.1376G>A | Nonsense | - | - | 1 |
| p.Trp679Ter | c.2037G>A | Nonsense | - | rs1377267685 | 1 |
| p.Trp801Ter | c.2402G>A | Nonsense | - | - | 1 |
| p.Tyr15Ter | c.45C>G | Nonsense | - | rs2082265760 | 1 |
| p.Val395TrpfsTer66 | c.1182del | Frameshift | - | - | 1 |
| p.Val906TrpfsTer11 | c.2712del | Frameshift | - | - | 1 |
|  | c.475+2del | Splice donor | - | - | 1 |
|  | c.475+2T>A | Splice donor | - | - | 1 |
|  | c.476-3C>G | splice_region_variant | - | - | 1 |
|  | c.1371-2A>G | Splice acceptor | - | - | 1 |

* indicates variants reported as somatic variants in the literature. All cDNA and protein changes are annotated according to the *ARMC5* transcript ENST00000268314.9.

**Supplementary Table 2.** *KDM1A* variants predicted to be potentially deleterious by in silico tools, but not reported as germline pathogenic or likely pathogenic in ClinVar/LOVD.

| **HGVS Consequence** | **Transcript Consequence** | **Variant type** | **ClinVar Variation ID** | **rsID** | **Allele count** |
| --- | --- | --- | --- | --- | --- |
| p.Arg196AsnfsTer6 | c.587_590del | Frameshift | - | - | 49 |
| p.Trp815Ter | c.2445G>A | Nonsense | - | rs1358867166 | 13 |
| p.Arg844Ter | c.2530C>T | Nonsense | - | - | 12 |
| p.Ile833TyrfsTer19 | c.2496_2497insT | Frameshift | - | - | 11 |
| p.Arg839PhefsTer14 | c.2510_2513dup | Frameshift | - | - | 7 |
| p.Ile833PhefsTer34 | c.2497del | Frameshift | - | - | 6 |
| p.Phe837SerfsTer29 | c.2510_2513del | Frameshift | - | - | 5 |
| - | c.1849_1867+39dup | Nonsense | - | rs1557596078 | 4 |
| p.Arg336Ter | c.1006C>T | Nonsense | - | rs1369524188 | 4 |
| p.Arg615Ter | c.1843C>T | Nonsense | - | rs1643495733 | 4 |
| p.Arg819Ter | c.2455C>T | Nonsense | 2757375 | rs746295431 | 4 |
| p.Gly623Ter | c.1867G>T | Nonsense | - | - | 4 |
| p.Leu854TrpfsTer29 | c.2561del | Frameshift | - | rs1643675950 | 4 |
| p.Lys480AsnfsTer12 | c.1440_1443del | Frameshift | - | rs763785057 | 4 |
| p.Arg839PhefsTer30 | c.2509_2513dup | Frameshift | - | - | 3 |
| p.Glu32Ter | c.94G>T | Nonsense | - | - | 3 |
| p.Lys246Ter | c.736A>T | Nonsense | - | - | 3 |
| p.Arg202Ter | c.604C>T | Nonsense | 2663425 | rs1341951118 | 2 |
| p.Arg750Ter | c.2248C>T | Nonsense | - | - | 2 |
| p.Cys751Ter | c.2253C>A | Nonsense | - | rs771833144 | 2 |
| p.Gln871ProfsTer54 | c.2611dup | Frameshift | - | - | 2 |
| p.Glu467Ter | c.1399G>T | Nonsense | - | rs766849199 | 2 |
| p.Leu651ArgfsTer4 | c.1952del | Frameshift | - | rs1242225620 | 2 |
| p.Pro869LeufsTer14 | c.2606del | Frameshift | 3657889 | rs1196220949 | 2 |
| p.Ser569AsnfsTer5 | c.1705_1706del | Frameshift | - | - | 2 |
| p.Trp576Ter | c.1727G>A | Nonsense | - | - | 2 |
| p.Val771GlyfsTer7 | c.2312_2313del | Frameshift | 2779015 | - | 2 |
| c.-5_41del | c.-5_41del | Loss-of-start-codon | - | - | 1 |
| c.-5_29del | c.-5_29del | Loss-of-start-codon | - | - | 1 |
| c.712-6A>G | c.712-6A>G | Splice region | - | rs767988319 | 1 |
| c.351+2T>A | c.351+2T>A | Splice donor | - | rs1641565510 | 1 |
| c.689_711+8del | c.689_711+8del | Splice donor | - | rs1642635701 | 1 |
| c.711+1G>T | c.711+1G>T | Splice donor | - | - | 1 |
| c.790+2T>A | c.790+2T>A | Splice donor | - | - | 1 |
| c.990+1G>T | c.990+1G>T | Splice donor | - | - | 1 |
| c.1548+1G>T | c.1548+1G>T | Splice donor | - | - | 1 |
| c.1622+1G>T | c.1622+1G>T | Splice donor | - | - | 1 |
| c.1734+1G>A | c.1734+1G>A | Splice donor | - | - | 1 |
| c.2298+2T>A | c.2298+2T>A | Splice donor | - | - | 1 |
| c.2445+1G>A | c.2445+1G>A | Splice donor | - | - | 1 |
| c.352-2A>G | c.352-2A>G | Splice acceptor | - | - | 1 |
| c.884-2A>G | c.884-2A>G | Splice acceptor | - | - | 1 |
| c.1323-1G>C | c.1323-1G>C | Splice acceptor | - | rs1404494656 | 1 |
| c.1414-1G>C | c.1414-1G>C | Splice acceptor | - | - | 1 |
| c.1735-2A>G | c.1735-2A>G | Splice acceptor | - | - | 1 |
| c.1868-1G>A | c.1868-1G>A | Splice acceptor | - | - | 1 |
| c.2056-2A>T | c.2056-2A>T | Splice acceptor | - | - | 1 |
| c.2171-2A>G | c.2171-2A>G | Splice acceptor | - | - | 1 |
| c.2299-2A>C | c.2299-2A>C | Splice acceptor | - | - | 1 |
| *c.352-1G>A | c.352-1G>A | Splice acceptor | 3366857 | - | 1 |
| *p.Arg271Ter | c.811C>T | Nonsense | 3366858 | - | 1 |
| p.Ala13GlyfsTer23 | c.36_37del | Frameshift | - | - | 1 |
| p.Ala312ValfsTer38 | c.931_934dup | Frameshift | - | - | 1 |
| p.Ala54ArgfsTer48 | c.160del | Frameshift | - | rs1314693338 | 1 |
| p.Ala729GlyfsTer16 | c.2185dup | Frameshift | - | - | 1 |
| p.Ala820LeufsTer10 | c.2458del | Frameshift | - | - | 1 |
| p.Ala846GlufsTer35 | c.2532_2538del | Frameshift | - | rs1643675069 | 1 |
| p.Ala846LysfsTer38 | c.2534_2535dup | Frameshift | - | - | 1 |
| p.Arg315Ter | c.943C>T | Nonsense | - | - | 1 |
| p.Arg550LysfsTer39 | c.1649_1652del | Frameshift | - | - | 1 |
| p.Arg61GlnfsTer64 | c.180_181del | Frameshift | - | - | 1 |
| p.Arg824GlufsTer6 | c.2470del | Frameshift | - | rs1643646435 | 1 |
| p.Arg848LysfsTer77 | c.2540dup | Frameshift | - | rs1261054895 | 1 |
| p.Asn142MetfsTer67 | c.425del | Frameshift | - | - | 1 |
| p.Asn611IlefsTer16 | c.1832del | Frameshift | - | - | 1 |
| p.Asn700GlnfsTer34 | c.2090_2097dup | Frameshift | - | - | 1 |
| p.Asp579GlyfsTer2 | c.1733dup | Frameshift | - | rs1297116846 | 1 |
| p.Asp745ValfsTer2 | c.2234del | Frameshift | - | - | 1 |
| p.Cys522AlafsTer50 | c.1564del | Frameshift | - | - | 1 |
| p.Cys689PhefsTer2 | c.2066_2067del | Frameshift | - | rs771234701 | 1 |
| p.Cys694Ter | c.2082C>A | Nonsense | - | - | 1 |
| p.Gln227ArgfsTer23 | c.679del | Frameshift | - | - | 1 |
| p.Gln533Ter | c.1597C>T | Nonsense | - | rs1643339431 | 1 |
| p.Gln656Ter | c.1966C>T | Nonsense | 2638479 | rs2124544754 | 1 |
| p.Gln657Ter | c.1969C>T | Nonsense | - | - | 1 |
| p.Gln871Ter | c.2611C>T | Nonsense | - | - | 1 |
| p.Glu103ArgfsTer23 | c.306dup | Frameshift | - | - | 1 |
| p.Glu106Ter | c.316G>T | Nonsense | - | - | 1 |
| p.Glu481AsnfsTer12 | c.1440del | Frameshift | - | rs758249030 | 1 |
| p.Glu57SerfsTer45 | c.169del | Frameshift | - | - | 1 |
| p.Glu604ArgfsTer6 | c.1809dup | Frameshift | - | - | 1 |
| p.Glu714GlyfsTer10 | c.2140dup | Frameshift | - | - | 1 |
| p.Glu825AsnfsTer16 | c.2472del | Frameshift | - | - | 1 |
| p.Gly100GlufsTer2 | c.299del | Frameshift | - | - | 1 |
| p.Gly100Ter | c.298G>T | Nonsense | - | - | 1 |
| p.Gly184Ter | c.550G>T | Nonsense | - | - | 1 |
| p.Gly584Ter | c.1750G>T | Nonsense | - | rs1482001729 | 1 |
| p.Gly594AlafsTer9 | c.1781del | Frameshift | - | - | 1 |
| p.Gly823Ter | c.2467G>T | Nonsense | - | rs750419196 | 1 |
| p.His273GlnfsTer15 | c.819_820del | Frameshift | - | rs753380832 | 1 |
| p.His508GlnfsTer11 | c.1524_1525del | Frameshift | - | - | 1 |
| p.Ile186PhefsTer34 | c.556_557del | Frameshift | - | - | 1 |
| p.Ile304ArgfsTer42 | c.911_918del | Frameshift | - | - | 1 |
| p.Ile627LysfsTer3 | c.1880del | Frameshift | - | - | 1 |
| p.Ile742MetfsTer2 | c.2226_2227del | Frameshift | - | - | 1 |
| p.Ile804ThrfsTer37 | c.2411del | Frameshift | 3371823 | - | 1 |
| p.Ile818GlyfsTer11 | c.2452_2455del | Frameshift | - | - | 1 |
| p.Leu545IlefsTer46 | c.1631_1632dup | Frameshift | - | rs1197604081 | 1 |
| p.Leu667Ter | c.1998_2002del | Frameshift | - | - | 1 |
| p.Leu727ThrfsTer18 | c.2178dup | Frameshift | - | rs1248004261 | 1 |
| p.Leu860TyrfsTer66 | c.2573_2576dup | Frameshift | - | rs1643676491 | 1 |
| p.Lys478AsnfsTer2 | c.1431_1432del | Frameshift | - | - | 1 |
| p.Lys5ArgfsTer97 | c.12del | Frameshift | - | - | 1 |
| p.Lys768Ter | c.2302A>T | Nonsense | - | - | 1 |
| p.Met97HisfsTer29 | c.288dup | Frameshift | - | - | 1 |
| p.Phe579CysfsTer21 | c.1736_1740del | Frameshift | - | rs1286556201 | 1 |
| p.Phe582Ter | c.1745_1746del | Frameshift | - | - | 1 |
| p.Phe694ValfsTer21 | c.2079dup | Frameshift | - | - | 1 |
| p.Phe702LeufsTer29 | c.2106del | Frameshift | - | - | 1 |
| p.Phe834TyrfsTer18 | c.2500_2501insA | Frameshift | - | - | 1 |
| p.Phe837GlnfsTer13 | c.2509_2513del | Frameshift | - | - | 1 |
| p.Pro154HisfsTer55 | c.459del | Frameshift | - | rs1357802822 | 1 |
| p.Pro47GlnfsTer55 | c.140del | Frameshift | - | - | 1 |
| p.Pro668ArgfsTer16 | c.2003_2004del | Frameshift | - | - | 1 |
| p.Pro765LeufsTer35 | c.2292del | Frameshift | - | - | 1 |
| p.Ser192Ter | c.575C>A | Nonsense | - | - | 1 |
| p.Ser319PhefsTer29 | c.954_955del | Frameshift | 2637430 | - | 1 |
| p.Ser3TrpfsTer33 | c.8_9del | Frameshift | - | - | 1 |
| p.Ser490LeufsTer3 | c.1469del | Frameshift | - | rs1447395807 | 1 |
| p.Ser569Ter | c.1706C>G | Nonsense | - | rs1303600379 | 1 |
| p.Ser572ProfsTer2 | c.1714_1715del | Frameshift | 3573678 | rs746906223 | 1 |
| p.Ser596ValfsTer13 | c.1786_1787del | Frameshift | - | rs1643494276 | 1 |
| p.Ser784LeufsTer12 | c.2351_2352del | Frameshift | - | - | 1 |
| p.Thr21ArgfsTer81 | c.60del | Frameshift | - | - | 1 |
| p.Thr27GlnfsTer75 | c.78del | Frameshift | - | - | 1 |
| p.Thr770LeufsTer30 | c.2308del | Frameshift | - | - | 1 |
| p.Trp241Ter | c.722G>A | Nonsense | - | - | 1 |
| p.Trp241Ter | c.723G>A | Nonsense | - | - | 1 |
| p.Trp555Ter | c.1665G>A | Nonsense | - | rs1435710328 | 1 |
| p.Trp670Ter | c.2010G>A | Nonsense | - | rs757593656 | 1 |
| p.Trp719Ter | c.2157G>A | Nonsense | - | - | 1 |
| p.Trp719Ter | c.2157del | Frameshift | - | - | 1 |
| p.Trp775Ter | c.2325G>A | Nonsense | - | rs1218474953 | 1 |
| p.Trp780Ter | c.2340G>A | Nonsense | - | - | 1 |
| p.Tyr262Ter | c.786_790del | Frameshift | - | - | 1 |
| p.Tyr415ThrfsTer6 | c.1243del | Frameshift | - | - | 1 |
| p.Val346SerfsTer2 | c.1036_1037del | Frameshift | - | - | 1 |
| p.Val402GlyfsTer6 | c.1204dup | Frameshift | 2011537 | rs1643221111 | 1 |
| p.Val764ThrfsTer14 | c.2289_2290del | Frameshift | - | - | 1 |
| p.Val89GlyfsTer37 | c.265dup | Frameshift | - | - | 1 |

* indicates variants reported as somatic variants in the literature. All cDNA and protein changes are annotated according to the *KDM1A* transcript ENST00000400181.9.

**Supplementary table 3.** Estimated carrier frequency of ARMC5 mutation per 100,000 population (95% CI) and standard error (SE) stratified by ancestry group and variant selection criteria

| Ancestry | ClinVar/LOVD (per 100k, 95% CI) | SE | Literature germline (per 100k, 95% CI) | SE | ClinVar/LOVD or Literature germline (per 100k, 95% CI) | SE | Literature somatic (per 100k, 95% CI) | SE | In silico* (per 100k, 95% CI) | SE | Any  (per 100k, 95% CI) | SE |
| --- | --- | --- | --- | --- | --- | --- | --- | --- | --- | --- | --- | --- |
| All | 11.6 (9.3–14.0) | 1.20 | 35.6 (31.4–39.7) | 2.10 | 37.5 (33.3–41.8) | 2.16 | 6.9 (5.1–8.8) | 0.93 | 35.3 (31.2–39.4) | 2.09 | 64.1 (58.5–69.6) | 2.82 |
| AFR | 21.3 (6.5–36.1) | 7.53 | 34.6 (15.8–53.4) | 9.60 | 40.0 (19.7–60.2) | 10.31 | 16.0 (3.2–28.8) | 6.52 | 53.3 (29.9–76.6) | 11.91 | 69.3 (42.6–95.9) | 13.58 |
| AMR | 6.7 (-2.6–15.9) | 4.71 | 66.6 (37.4–95.8) | 14.89 | 73.3 (42.7–103.9) | 15.62 | 6.7 (-2.6–15.9) | 4.71 | 50.0 (24.7–75.2) | 12.90 | 119.9 (80.8–159.1) | 19.98 |
| ASJ | 0.0 (0.0–0.0) | 0.00 | 6.8 (-6.5–20.0) | 6.75 | 6.8 (-6.5–20.0) | 6.75 | 0.0 (0.0–0.0) | 0.00 | 27.0 (0.5–53.5) | 13.51 | 27.0 (0.5–53.5) | 13.51 |
| EAS | 13.4 (-1.8–28.5) | 7.72 | 106.9 (64.2–149.7) | 21.81 | 111.4 (67.7–155.0) | 22.26 | 8.9 (-3.4–21.3) | 6.30 | 75.7 (39.7–111.7) | 18.36 | 173.7 (119.3–228.2) | 27.80 |
| FIN | 25.0 (7.7–42.3) | 8.83 | 25.0 (7.7–42.3) | 8.83 | 31.2 (11.9–50.6) | 9.87 | 15.6 (1.9–29.3) | 6.98 | 59.3 (32.7–86.0) | 13.61 | 74.9 (45.0–104.9) | 15.29 |
| MID | 0.0 (0.0–0.0) | 0.00 | 0.0 (0.0–0.0) | 0.00 | 0.0 (0.0–0.0) | 0.00 | 0.0 (0.0–0.0) | 0.00 | 33.0 (-31.7–97.6) | 32.99 | 33.0 (-31.7–97.6) | 32.99 |
| NFE | 10.8 (8.2–13.5) | 1.36 | 34.4 (29.7–39.1) | 2.41 | 35.8 (30.9–40.6) | 2.46 | 6.3 (4.3–8.3) | 1.03 | 30.8 (26.4–35.3) | 2.29 | 58.6 (52.5–64.8) | 3.15 |
| SAS | 15.4 (4.0–26.8) | 5.81 | 17.6 (5.4–29.7) | 6.21 | 17.6 (5.4–29.7) | 6.21 | 8.8 (0.2–17.4) | 4.39 | 39.5 (21.3–57.8) | 9.31 | 48.3 (28.1–68.5) | 10.30 |
| RMI | 6.4 (-2.5–15.3) | 4.52 | 32.0 (12.2–51.8) | 10.12 | 35.2 (14.4–56.0) | 10.61 | 0.0 (0.0–0.0) | 0.00 | 28.8 (10.0–47.6) | 9.60 | 60.8 (33.5–88.1) | 13.94 |

Abbreviations: 95% CI: 95% confidence interval of proportion (per 100,000); SE: standard error of proportion; AFR: African/African American; AMR: Admixed American; ASJ: Ashkenazi Jewish; EAS: East Asian; FIN: Finnish; MID: Middle Eastern; NFE: Non-Finnish European; SAS: South Asian; RMI: Remaining.
* Variants identified by in silico criteria were defined as nonsense, frameshift, or loss-of-start-codon variants, or splice-site variants with a SpliceAI score ≥ 0.8, CADD score > 20, and |pangolin score| > 0.2, excluding variants with an allele frequency > 10⁻⁴.

**Supplementary table 4.** Sensitivity analysis of estimated carrier prevalence ARMC5 variants per 100,000 (95% CI) and standard error (SE), stratified by ancestry and variant selection criteria

| Ancestry | Strict criteria (excluding variants with AF >1×10⁻⁵) (per 100k, 95% CI) | SE | Liberal criteria (excluding variants with AF >1×10⁻⁵) (per 100k, 95% CI) | SE | Variants with evidence of second somatic hit (per 100k, 95% CI) | SE | Variants with functional evidence (per 100k, 95% CI) | SE | Strict criteria (excluding variants with AF >1×10⁻⁵) (per 100k, 95% CI) | SE | Liberal criteria (excluding variants with AF >1×10⁻⁵) (per 100k, 95% CI) | SE |
| --- | --- | --- | --- | --- | --- | --- | --- | --- | --- | --- | --- | --- |
| All | 16.5 (13.7–19.3) | 1.43 | 43.0 (38.5–47.5) | 2.31 | 17.7 (14.8–20.6) | 1.48 | 3.0 (1.8–4.2) | 0.61 | 16.5 (13.7–19.3) | 1.43 | 43.0 (38.5–47.5) | 2.31 |
| AFR | 34.6 (15.8–53.4) | 9.6 | 63.9 (38.4–89.5) | 13.04 | 26.6 (10.1–43.1) | 8.42 | 8.0 (-1.1–17.0) | 4.61 | 34.6 (15.8–53.4) | 9.6 | 63.9 (38.4–89.5) | 13.04 |
| AMR | 10.0 (-1.3–21.3) | 5.77 | 56.6 (29.7–83.5) | 13.73 | 6.7 (-2.6–15.9) | 4.71 | 0.0 (0.0–0.0) | 0 | 10.0 (-1.3–21.3) | 5.77 | 56.6 (29.7–83.5) | 13.73 |
| ASJ | 6.8 (-6.5–20.0) | 6.75 | 27.0 (0.5–53.5) | 13.51 | 0.0 (0.0–0.0) | 0 | 0.0 (0.0–0.0) | 0 | 6.8 (-6.5–20.0) | 6.75 | 27.0 (0.5–53.5) | 13.51 |
| EAS | 13.4 (-1.8–28.5) | 7.72 | 75.7 (39.7–111.7) | 18.36 | 17.8 (0.4–35.3) | 8.91 | 0.0 (0.0–0.0) | 0 | 13.4 (-1.8–28.5) | 7.72 | 75.7 (39.7–111.7) | 18.36 |
| FIN | 31.2 (11.9–50.6) | 9.87 | 74.9 (45.0–104.9) | 15.29 | 25.0 (7.7–42.3) | 8.83 | 15.6 (1.9–29.3) | 6.98 | 31.2 (11.9–50.6) | 9.87 | 74.9 (45.0–104.9) | 15.29 |
| MID | 0.0 (0.0–0.0) | 0 | 33.0 (-31.7–97.6) | 32.99 | 0.0 (0.0–0.0) | 0 | 0.0 (0.0–0.0) | 0 | 0.0 (0.0–0.0) | 0 | 33.0 (-31.7–97.6) | 32.99 |
| NFE | 15.6 (12.4–18.8) | 1.63 | 38.5 (33.5–43.5) | 2.55 | 18.3 (14.9–21.8) | 1.76 | 2.4 (1.1–3.6) | 0.63 | 15.6 (12.4–18.8) | 1.63 | 38.5 (33.5–43.5) | 2.55 |
| SAS | 15.4 (4.0–26.8) | 5.81 | 46.1 (26.4–65.8) | 10.06 | 15.4 (4.0–26.8) | 5.81 | 2.2 (-2.1–6.5) | 2.2 | 15.4 (4.0–26.8) | 5.81 | 46.1 (26.4–65.8) | 10.06 |
| RMI | 12.8 (0.3–25.3) | 6.4 | 38.4 (16.7–60.1) | 11.08 | 12.8 (0.3–25.3) | 6.4 | 3.2 (-3.1–9.5) | 3.2 | 12.8 (0.3–25.3) | 6.4 | 38.4 (16.7–60.1) | 11.08 |

Abbreviations: 95% CI: 95% confidence interval of proportion (per 100,000); SE: standard error of proportion; AFR: African/African American; AMR: Admixed American; ASJ: Ashkenazi Jewish; EAS: East Asian; FIN: Finnish; MID: Middle Eastern; NFE: Non-Finnish European; SAS: South Asian; RMI: Remaining.

**Supplementary table 5.** Estimated carrier frequency of KDMA1 mutation per 100,000 population (95% CI) and standard error (SE) stratified by ancestry group and variant selection criteria

| Ancestry | ClinVar/LOVD (per 100k, 95% CI) | SE | Nonsense/frameshift/loss-of-start-codon variants  (per 100k, 95% CI) | SE | Splice-site variants* (per 100k, 95% CI) | SE | Any (per 100k, 95% CI) | SE |
| --- | --- | --- | --- | --- | --- | --- | --- | --- |
| All | 0.2 (-0.1–0.6) | 0.18 | 31.2 (27.4–35.1) | 1.97 | 2.6 (1.5–3.7) | 0.57 | 33.8 (29.8–37.8) | 2.05 |
| AFR | 0.0 (0.0–0.0) | 0 | 16.0 (3.2–28.8) | 6.52 | 0.0 (0.0–0.0) | 0 | 16.0 (3.2–28.8) | 6.52 |
| AMR | 0.0 (0.0–0.0) | 0 | 23.3 (6.0–40.6) | 8.81 | 13.3 (0.3–26.4) | 6.66 | 36.6 (15.0–58.3) | 11.05 |
| ASJ | 0.0 (0.0–0.0) | 0 | 27.0 (0.5–53.5) | 13.51 | 0.0 (0.0–0.0) | 0 | 27.0 (0.5–53.5) | 13.51 |
| EAS | 0.0 (0.0–0.0) | 0 | 22.3 (2.8–41.8) | 9.96 | 0.0 (0.0–0.0) | 0 | 22.3 (2.8–41.8) | 9.96 |
| FIN | 0.0 (0.0–0.0) | 0 | 6.2 (-2.4–14.9) | 4.42 | 0.0 (0.0–0.0) | 0 | 6.2 (-2.4–14.9) | 4.42 |
| MID | 0.0 (0.0–0.0) | 0 | 66.0 (-25.4–157.4) | 46.64 | 0.0 (0.0–0.0) | 0 | 66.0 (-25.4–157.4) | 46.64 |
| NFE | 0.3 (-0.1–0.8) | 0.24 | 35.3 (30.5–40.0) | 2.44 | 2.5 (1.3–3.8) | 0.66 | 37.8 (32.8–42.8) | 2.53 |
| SAS | 0.0 (0.0–0.0) | 0 | 28.5 (13.0–44.1) | 7.92 | 0.0 (0.0–0.0) | 0 | 28.5 (13.0–44.1) | 7.92 |
| RMI | 0.0 (0.0–0.0) | 0 | 16.0 (2.0–30.0) | 7.15 | 6.4 (-2.5–15.3) | 4.52 | 22.4 (5.8–39.0) | 8.46 |

Abbreviations: 95% CI: 95% confidence interval of proportion (per 100,000); SE: standard error of proportion; AFR: African/African American; AMR: Admixed American; ASJ: Ashkenazi Jewish; EAS: East Asian; FIN: Finnish; MID: Middle Eastern; NFE: Non-Finnish European; SAS: South Asian; RMI: Remaining.
* Defined as splice-site variants with a SpliceAI score ≥ 0.8, CADD score > 20, and |pangolin score| > 0.2. Variants with an allele frequency > 10⁻⁴ were excluded.
